# Freud’s 1926 conjecture is confirmed: evidence from the dorsal periaqueductal gray in mice that human psychological defense against internal instinctual threat evolved from animal motor defense against external predatory threat

**DOI:** 10.3389/fpsyg.2024.1427816

**Published:** 2024-09-24

**Authors:** Paul J. Schwartz

**Affiliations:** Section on Ego Mechanics, Cincinnati Psychoanalytic Institute, Cincinnati, OH, United States

**Keywords:** Freud, defense, instinct, periaqueductal gray, phylogenetic, fear, computational, evolution

## Abstract

In 1926, Freud famously conjectured that the human ego defense of repression against an *internal* instinctual threat evolved from the animal motor defense of flight from an *external* predatory threat. Studies over the past 50 years mainly in rodents have investigated the neurobiology of the fight-or-flight reflex to external threats, which activates the emergency alarm system in the dorsal periaqueductal gray (dPAG), the malfunction of which appears likely in panic and post-traumatic stress disorders, but perhaps also in some “non-emergent” conditions like social anxiety and “hysterical” conversion disorder. Computational neuroscience studies in mice by Reis and colleagues have revealed unprecedented insights into the dPAG-related neural mechanisms underlying these evolutionarily honed emergency vertebrate defensive functions (e.g., explore, risk assessment, escape, freeze). A psychoanalytic interpretation of the Reis studies demonstrates that Freud’s 1926 conjecture is confirmed, and that internal instinctual threats alone can also set off the dPAG emergency alarm system, which is regulated by 5-HT_1A_ and CRF-1 receptors. Consistent with current psychoanalytic and neurobiologic theories of panic, several other of the primitive components of the dPAG alarm system may also have relevance for understanding of the unconscious determinants of impaired object relationships (e.g., avoidance distance). These dPAG findings reveal (1) a process of “evolution *in situ*,” whereby a more sophisticated dPAG ego defense is seen evolving out of a more primitive dPAG motor defense, (2) a dPAG location for the phylogenetically ancient kernel of Freud’s Ego and Id, and (3) a Conscious Id theory that has been conclusively invalidated.

## Introduction

A century ago, after more than 30 years of pioneering psychoanalytic work, Freud published his theoretical synthesis, “The Ego and the Id” (Freud, 1923), describing his metapsychological model of the mental apparatus. This model has since served as a guide for psychoanalytically-based treatments for various types of debilitating emotional disorders (Leichsenring, 2005). Prospective (Kernberg et al., 1972; Kantrowitz et al., 1987), meta-analytic (de Maat et al., 2013), and follow-up studies (Leuzinger-Bohleber et al., 2003) have clearly demonstrated the effectiveness of psychoanalytic treatments for carefully selected patients who are both healthy enough as well as sick enough to warrant such an extended, intensive, and ambitious treatment. Freud succinctly summed up his treatment method—which aims to loosen Ego defenses and allow the emergence of threatening unconscious id-related material into consciousness and thereby allow for its therapeutic resolution (Waldron et al., 2015)—in his oft-quoted statement, “Where id was, there ego shall be” (Freud, 1933). Some compelling modern neuroscientific evidence supports the metapsychological construct validity of Freud’s Ego-Id model including the general brain substrates of its defensive operations (Watt, 1990; Northoff and Boeker, 2006; Carhart-Harris and Friston, 2010; Northoff and Scalabrini, 2021). However, a direct linkage between the specific neuronal substrates underlying a specific instinct-defense operation has not yet been demonstrated in either animals or humans.

Notwithstanding the embedded nature of Freud’s influence in most of the world’s cultural psychology, his metapsychological model of the mental apparatus as well as the practice of psychoanalysis are becoming imperiled by forces both internal and external to psychoanalysis. Internally, a new and quite influential, but controversial neuropsychoanalytic model of the mind is becoming ascendant and is purported to be based on more rigorous and modern neuroscientific findings and principles (Solms, 2013; Solms, 2021a; Solms, 2021b). Externally, societal and economic forces and psychopharmacologic advances have rendered this lengthy, inconvenient, and expensive psychoanalytic treatment—which may nevertheless be uniquely effective for some (Leichsenring, 2005)—unavailable to most. These internal and external threats to the Freudian metapsychological model of the mind mirror the internal (id instincts) and external (objects in reality) threats to the very survival of Freud’s adaptive Ego itself. “Thus the ego is fighting on two fronts: it has to defend its existence against an *external* world which threatens it with annihilation as well as against an *internal* world that makes excessive demands” (Freud, 1938).

In 1926, Freud conjectured that the psychological Ego defenses that humans employ to manage imminent threats from the *internal* world (i.e., instincts) evolved phylogenetically from the more primitive motor defenses that animals employ to manage imminent threats from the *external* world (i.e., predators). “The defense against an unwelcome *internal* process will be modeled upon the defense adopted against an *external* stimulus, that the ego wards off internal and external dangers alike along identical lines. In the case of external danger, the organism has recourse to attempts at flight… Repression is an equivalent of this attempt at flight” (Freud, 1926; Freud, 1938).

In his lifetime, Freud could not have been aware that some of the critical neural components of such ancient and evolutionarily conserved, emergency alarm defensive circuits (e.g., fight or flight) are housed in the upper brainstem periaqueductal gray (PAG) (Cisek, 2021). The PAG has become intensely investigated because of its potential role various states of debilitating anxiety and fear (e.g., panic, post-traumatic stress), which may unfortunately be quite intransigent to currently available pharmacological treatments (Williams et al., 2022; Guaiana et al., 2023) and cognitive-based therapies (Pompoli et al., 2016; Lewis et al., 2020), and which are characterized in neuroimaging studies of humans by heightened PAG activation following exposure to proximate threats such as exposure to a live tarantula (Mobbs et al., 2007; Mobbs et al., 2020). It should be noted however that heightened PAG activation has also been observed in a variety of other fear-anxiety states that are not typically considered “emergent” [e.g., social anxiety (Arnold Anteraper et al., 2014) and conversion disorder (Aybek and Vuilleumier, 2016)]. Animal studies indicate that the dorsal subregion of the PAG (dPAG) is the PAG region that is most critically involved with the instinct to flee (variably termed “flight” or “escape”) from a terrifying threat (Graeff, 1981; Brandão et al., 1982). Conceivably, a greater psychoanalytic understanding of the dPAG’s phylogenetically preserved instinctual and defensive functions may reveal important mechanistic and therapeutic insights into such debilitating psychopathological clinical states that are potentially related to the dPAG’s emergency alarm system. Further, any legitimate metapsychological model of the mind must be able to account for any such findings in the dPAG.

The present conceptual analysis provides a neuropsychoanalytic and metapsychological framework for understanding the phylogenetic underpinnings of our basic instincts and defenses that were predicted in Freud’s conjecture of 1926.

## The upper brainstem dPAG

The PAG is an evolutionarily ancient neural organization that is found with mostly homologous phylogenetic anatomic locations and organizations, molecular profiles, and afferent and efferent neural connections, including in the one of the oldest known living vertebrates, the jawless, eel-like lamprey fish (Olson et al., 2017; Miyashita et al., 2021). The PAG is a tube-shaped mass of neuronal cell bodies that envelopes the cerebral aqueduct, which is a conduit for cerebrospinal fluid flow and connects the third ventricle at the level of the midbrain to the fourth ventricle at the level of the pons. The PAG can be anatomically and functionally divided into different cellular columns that run parallel along the length of the PAG— the dorsomedial, dorsolateral, lateral, and ventrolateral columns. The dorsomedial and dorsolateral columns are often combined and studied together in the more broadly defined dPAG (Gomes and Nunes-de-Souza, 2009; Reis et al., 2021a; Reis et al., 2021b). Each of these PAG subregions mediates with some specificity their various functions, such as defensive (e.g., explore, flight) and autonomic (e.g., parasympathetic, sympathetic) responses to threat (Bandler et al., 1991; Carrive, 1993; Bandler and Shipley, 1994; Brandão et al., 1994; Bandler et al., 2000; Silva and McNaughton, 2019; Reis et al., 2023).

In animals, the dPAG has probably been the most extensively studied of all the PAG subregions. The dPAG is primarily involved in mediating the animal’s emergency behavioral responses to proximate and imminent external dangers (Deng et al., 2016; Tovote et al., 2016; Evans et al., 2018; Estaban Masferrer et al., 2020; Reis et al., 2021a). Phylogenetic threats (Panksepp, 1998) (e.g., cat odor, snakes) that have never previously been encountered in their lifetimes by either rodents (Dielenberg et al., 2001) or by non-human primates (Montardy et al., 2021) are instinctively “remembered” and trigger immediate and intense terror, the instinct to flee, and heightened neuronal activation of the dPAG. Such PAG-mediated instinctual fear and terror responses have also been observed in fMRI studies of humans, especially when the external threat is perceived as close, and the escape must be initiated rapidly and instinctively (Mobbs et al., 2007; Mobbs et al., 2010; Mobbs et al., 2020).

An influential theory posits that there is a spectrum of fear/anxiety, such that pre-encounter generalized anxiety occurs when no external threat has been clearly identified, post-encounter fear/anxiety occurs when an external threat has been perceived and identified, and *circa*-strike terror occurs when the external threat has become too proximate and possibly life-threatening (Fanselow and Lester, 1988; Fanselow, 1994; Perusini and Fanselow, 2015). Consistent with these theories, the dPAG has been implicated in both animal and human studies in the emergency “*circa*-strike” terror that is pathologically activated in both panic attacks (Deakin and Graeff, 1991; Graeff and Del-Ben, 2008) and post-traumatic stress disorder (Adamec et al., 2012; Rabellino et al., 2016). However, as noted above, heightened PAG activation has also been observed in an increasing number of other fear/anxiety states that may not seem quite so “emergent,” including social anxiety (Arnold Anteraper et al., 2014), “hysterical” conversion disorders [a.k.a. ‘la belle indifference’ in functional neurological deficit disorders (Aybek and Vuilleumier, 2016)], and anxious temperament and depression (Kalin, 2017), suggesting that unconscious terror—even in the absence of a clearly identifiable external threat—may indeed activate this emergency alarm system. The extent to which the pathological dPAG hyperactivation in these various anxiety-, fear-, and mood-related conditions—which can at times be quite pharmacologically treatment-refractory to all currently available psychotropic medications—resolve following successful psychoanalytic treatment has not been studied.

## dPAG-mediated defensive behaviors of mice to innate and contextual threats

Reis and colleagues have conducted a remarkable and exquisite series of experiments that have revealed the distinct neuronal ensembles underlying aspects of some of the most fundamental of all vertebrate defensive survival behaviors (Reis et al., 2021a; Reis et al., 2021b). In one of their experiments, small implants containing hundreds of tiny microscopes were surgically positioned into the dPAGs of 8 mice (C57BL/6 J), and the fluctuating firing activities of their dPAG neurons were documented by the corresponding fluctuations in calcium fluorescence during the mice’s various behaviors throughout the experiment. Each mouse was placed into a rectangular cage that on consecutive days contained at one end, (1) a restrained predatory rat (“rat assay”), (2) a shock grid (“fear acquisition assay”), and (3) no rat and no shock grid (“fear retrieval assay”). The goal was to see whether any distinct neuronal ensemble profiles could be identified that specifically coded for the mice’s various defensive behaviors, and if so, whether these same neuronal substrates would remain uniform across the 3 different assays on the 3 consecutive days. If so, such persistence would indicate the development of contextual fear conditioning despite the absence of any overt threat—with important relevance, for example, to PTSD.

Reis and colleagues discovered that there were 4 statistically distinct neuronal ensembles in the dPAG, the activity of each of which selectively increased during the behavioral execution of the 4 respective, operationally-defined, manifest defensive behaviors, which tended to unfold in a cyclical sequence as follows: (1) “approach” (deliberatively approach and explore the rat), (2) “stretch” (flatten out and take a stealthy multisensory risk assessment of the rat), (3) “escape” (initiate immediate emergency flight from the rat) and (4) “freeze” (once retreated to a safe distance, hold still, chill down, stay undetected, and take sensory stock). These 4 defensive states were often interspersed by “non-behavioral” states—characterized as alert behavioral intervals during which no operationally defined manifest defensive states were exhibited.

**Figure 1:**
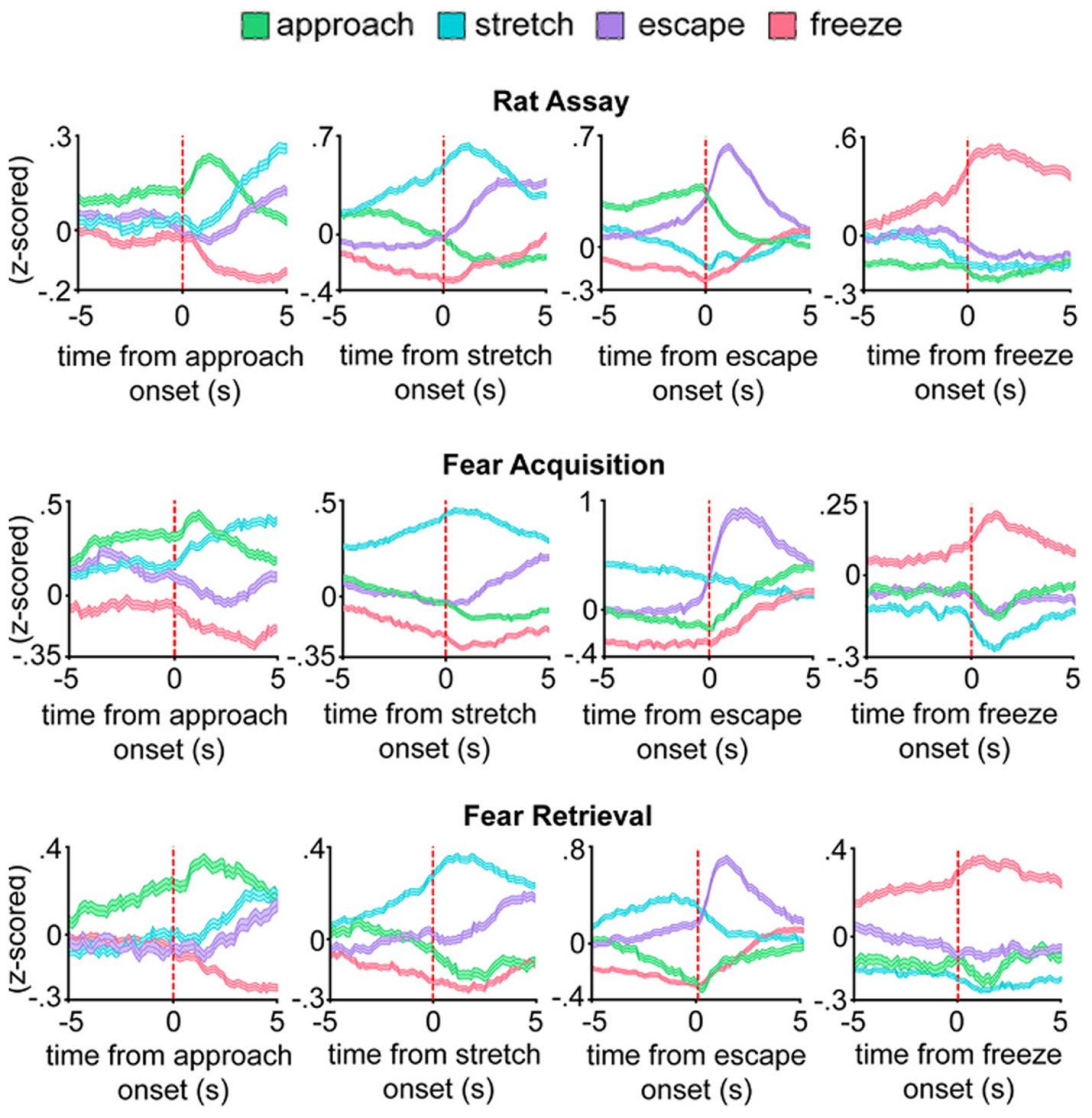
Freudian Ego and Id encoded in the neuronal ensembles of the dPAG. The neuronal ensemble firing rates for each of the 4 defensive behaviors ("approach," "stretch," "escape," and "freeze") are shown for each of the 3 different assays. Data for each defensive state are averaged and aligned to the onset of the operationally defined manifest defensive behavior, along with the remaining latent defensive/instinctual profiles for each manifest defensive behavior. Top 4 graphs: Innate threat, predatory rat present (Rat Assay). Middle 4 graphs: Shock grid, no rat present (Fear Acquisition Assay). Bottom 4 graphs: Neutral grid, no shock or rat present (Fear Retrieval Assay). See text for further explanation. Figure reproduced with permission from Reis et al. (2021a), Shared Dorsal Periaqueductal Gray Activation Patterns during Exposure to Innate and Conditioned Threats, Journal of Neuroscience 41(25):5399:5420, according to the Creative Commons Attribution 4.0 International License.

Figure 1 depicts the firing rate profiles for each of the 4 distinct dPAG ensembles during each of the 3 different assays. Because ‘distance to threat’ represents the overwhelmingly major correlate of dPAG neuronal activity (Perusini and Fanselow, 2015; Deng et al., 2016; Estaban Masferrer et al., 2020; Reis et al., 2021a) and could potentially statistically drown out any background defense-related neuronal signals, distance to threat was regressed out of the neuronal firing rate data for Figure 1, revealing the underlying signature traces of the 4 neuronal ensembles. Thus, across the 3 experimental assays, each of the 4 respective defensive behavioral states was characterized by its own dominant (highest) neuronal ensemble firing rate profile, as well as by its own unique array of 3 subordinate (lower) neuronal ensemble firing rate profiles—which nevertheless were dynamically not static and could conceivably reflect the influence of physiologically meaningful but latent “microstates” within each of the 4 manifest defensive behaviors (Signoret-Genest et al., 2023).

In addition to discovering that the 4 distinct defensive behaviors (“approach,” “stretch,” “escape,” and “freeze”) were each associated with their own statistically distinct set of 4 dPAG neuronal ensemble profiles (1 dominant and 3 subordinate), the authors also made the important discovery that for each of the 4 defensive behaviors, the rank order of their 4 ensemble profiles was essentially conserved across the 3 different assays, which were characterized by 3 very different types of contextual threats [i.e. innate (“rat”), shock (“fear acquisition”), and conditioned (“fear retrieval”)]. However, on closer inspection, there was one conspicuous exception to these 12 uniform rank orders in these assay-specific defensive profiles—that being during the 5-s interval preceding “escape” onset in the rat assay (i.e., in the presence of the rat), when “approach” levels were clearly high and dominant, whereas in both the “fear acquisition” and “fear retrieval “assays, “stretch” levels were clearly dominant. Such a clear and conspicuous reversal of rank order was observed only during “pre-escape” in the rat assay and was not observed in any of the other of the 11 experimental conditions, and hence begs for an explanation.

## When exploration becomes intolerably dangerous for timid mice

These considerations indicate that of the 4 defensive behaviors (“approach,” “stretch,” “escape,” and “freeze”), only “pre-escape” was differentially affected by the 3 different assays. That is, in the presence of the rat (i.e., “rat assay”), just prior to the “escape,” “approach” levels were high, whereas in the “fear acquisition” and “fear retrieval” assays, just prior to “escape,” “approach” levels were low, and instead “stretch” levels were high. These high levels of “pre-escape” “approach” were mostly due to high levels of “approach” during “non-behavioral” episodes just prior to “escape,” that is, when the mice were not exhibiting any overt motoric defensive behavior (see Figures 1D,F from Reis et al., 2021a). In their companion study, using the ‘elevated plus maze’ in an approach-avoidance experimental paradigm (Reis et al., 2021b), Reis and colleagues have further demonstrated highly significant and equal (slope) correlations between the proportions of time that a given mouse (1) “approaches” the rat and (2) “explores” the riskier exposed open arms of the ‘elevated plus maze,’ indicating that the “approach” and “explore” neuronal ensembles were essentially the same ensembles. Therefore, from hereafter, “approach” will be referred to as “explore.”

Thus, the high “explore” levels prior to “escape” in the “rat assay” (“innate condition”) indicate that the presence of the live rat induced a high level of activation of this “explore” instinct in mice, and yet this high level of “explore” was not enacted into any specific defensive motoric behavior, but rather has remained latent and “non-behavioral” in some manner, i.e., either “explore” was felt consciously but not acted upon, or “explore” was completely unfelt (subconscious or unconscious) and not acted upon. Additionally, prior to “escape” in the absence of the live predatory rat in the “fear acquisition” and “fear retrieval “assays, “explore” remains low and has not at all been activated. Thus, from a psychoanalytic perspective, it is fair to say that, just prior to “escape” and only in the “rat assay,” these timid mice have activated some type of affectively (inquisitive “explore”) and informationally (the rat is large and fierce but is hopefully neutralized) meaningful “object relation” with the restrained predatory rat.

In the presence of the predatory rat, when the mice’s increasing instinctual intrigue reaches a critical threshold, emergency “escape” is triggered (independent of the mice’s distance to the rat), their high levels of “explore” become immediately suppressed (or repressed), and they do a 180° and run for their lives, presumably with nothing else but survival on their minds. That is, it seems incompatible that mice could simultaneously consciously feel *both* (1) the terror of being cannibalized, and (2) the desire for elective, deliberative, and inquisitive “explore” while they are enacting their emergency survival “escape.” By contrast, in the absence of an “threatening object” in the “fear acquisition” and “fear retrieval” assays, “escape” is initiated precisely when “escape” has overtaken “stretch,” as if some hint of an unknown threat has been detected. Thus, if the “pre-escape” “explore” instinct was ever consciously felt, following the initiation of “escape,” the “explore” instinct becomes no longer consciously felt and has been defensively relegated to a some dynamically subconscious, incompletely compartmentalized, temporary holding area somewhere in the mental apparatus within the dPAG of the mouse.

## Freud’s 1926 conjecture: ego defense (repression) against internal instinctual threat evolves from motor defense (flight) against external predatory threat

The above considerations indicate that in mice, an increasing “explore” instinctual cathexis to the restrained predatory rat can become intolerably threatening to their rudimentary ego, which then simultaneously initiates the execution of defensive motor “escape” (flight) and immediate and rapid suppression (or repression) of the “explore” instinct into a temporary, incompletely developed and compartmentalized, subconscious holding area of the dPAG-sector of the mental apparatus of mice. These findings indicate that these timid mice initiate defensive “escape” not only when the external predatory threat has become proximate and imminent (Blanchard et al., 1986; Fanselow and Lester, 1988), but also “neurotically” when their own internal “explore” instinctual levels have become too proximate, imminent, and threatening to their rudimentary dPAG survival egos. During the course of evolution, an emergency “motor flight from predator” reflex is giving rise to an emergency “ego repression of instinct” reflex.

Freud’s 1926 conjecture about the evolution of human ego defense against internal instincts from phylogenetically ancient animal motor defense against external predators in the wild appears to be confirmed. “The defense against an unwelcome *internal* process will be modeled upon the defense adopted against an *external* stimulus, that the ego wards off internal and external dangers alike along identical lines. In the case of external danger, the organism has recourse to attempts at flight… Repression is an equivalent of this attempt at flight” (Freud, 1926; Freud, 1938). From an evolutionary perspective, such a duplication, remodeling, and repurposing of basic neuronal circuit modules in order to acquire more evolutionarily adaptive complex behavioral traits is the focus of ongoing theoretical and experimental research (Tosches, 2017; Barrett and Finlay, 2018; Seoane, 2020) and has been specifically investigated in relation to the evolution of dPAG defensive functions in non-human primates (Chang et al., 2013).

These dPAG-mediated motor and ego defenses in mice presumably reflect some past phylogenetic speciation “event” about 85 million years ago (Springer and Murphy, 2007) when shrew-like ancestors common to both mice and men diverged along different phylogenetic trajectories that led to their very different brain sizes and dPAG-mediated repertoires of defensive and psychological behaviors (e.g., sublimination and symbolism in humans). Similar phylogenetic adaptive considerations account for the allometric correlations between brain association area size and measures of behavioral innovation (e.g., tool use) in both primates and birds (Lefebvre et al., 2004). The finding that a dPAG psychological defense appears to be in the process of “budding off” from a dPAG motor defense thus precisely locates the neuroanatomic evolutionary history of a behavioral trait, and can be rightly designated as an example of “evolution *in situ*,” i.e. a process that is observed ‘in its place of origin’ at an early stage of evolutionary developmental differentiation and speciation (Rice and Pfennig, 2007).

## The neurobiology and psychodynamics of the dPAG instinct-repression reaction surface

Bill Deakin and Frederico Graeff, studying rats in the ‘elevated T-maze,’ formulated an evolutionary and neurobiological theory of aberrant serotonergic functioning of the dPAG emergency alarm system in the genesis of panic disorder (Deakin and Graeff, 1991). Robert and Caroline Blanchard, studying mice in a variety of experimental paradigms (Blanchard et al., 1993; Blanchard et al., 2001), formulated several evolutionary theories related to various aspects of rodent defenses (including risk assessment and flight) and their relevance to a broad range of human defenses and emotional disorders including panic (Blanchard and Blanchard, 1989; Blanchard et al., 2011; Blanchard, 2017). Watt and Panksepp (2009) and Panksepp (2011) have also formulated an integrated evolutionary neurobiological-psychodynamic theory of depression, separation distress, and panic, involving the CRF, opioids, oxytocin, and cholinergic systems in the dPAG. In particular, opioids interacting with serotonin receptors in the dPAG are importantly involved in mitigating the separation distress and panic that can be associated with flight responses, i.e., in the dPAG’s defensive mitigation the intensity of the emergency emotional response (Watt and Panksepp, 2009; Graeff, 2017). Of note here, however, is that each of these neurobiological animal models avoids any mention of defense against *unconscious* affect or instinct, and instead formulates that human defenses are mostly deployed against the threat from external *conscious* dangers and the urgent need to search for hiding places and escape routes (Blanchard et al., 2011).

Donald Klein, studying humans with panic disorder, formulated a “not conscious” CO_2_ sensitivity/suffocation theory of the emergency alarm system (Klein, 1993), which was later linked psychodynamically to separation anxiety (Preter and Klein, 2008) and located mechanistically to the dPAG (Schimitel et al., 2012). Busch and Milrod, studying patients with panic disorder, formulated a psychoanalytic theory of panic which holds that panic arises from defenses against *unconscious* emotional conflicts involving separation and autonomy (Busch et al., 2009). All of the above formulations of neurobiologic sensitivity and unconscious conflicts regarding separation and autonomy are consistent with the present formulation that ego repression may thwart any instinct to “explore” the world that is perceived as threatening.

The quantitative neurobiological aspects of “explore,” “escape,” and “fear/anxiety” in mice have been studied intensively over the past 50 years employing various experimental paradigms, mainly including the Mouse Defense Test Battery (MDTB) (Griebel et al., 1996; Blanchard et al., 1998; Blanchard et al., 2003), the ‘elevated plus maze’ (EPM) (Graeff et al., 1996; Gomes and Nunes-de-Souza, 2009; Reis et al., 2021b), and the Rat Exposure Test (RET) (Yang et al., 2004; Pobbe et al., 2011; Tovote et al., 2016). Studies employing these three experimental paradigms have been very influential in guiding the development of effective anxiolytic medications for humans (Griebel and Holmes, 2013). Although there appear to be species differences between mice and rats in their dorsal raphe presynaptic 5-HT_1A_ receptor regulation of dPAG serotonergic function (Pobbe et al., 2011), because dPAG 5-HT_1A_ receptors have been by far the most extensively studied of all the dPAG receptors in rodents including mice, only studies involving the acute intra-dPAG administration of 5-HT_1A_ receptor ligands in mice are reviewed here. In addition, studies involving intra-dPAG infusion of corticotropin releasing factor (CRF) receptor ligands in mice will also be reviewed.

In mice in the EPM (no predator), intra-dPAG infusion of the 5-HT_1A_ receptor agonist 8-OH-DPAT did not affect pre-encounter “explore” or “fear/anxiety” behaviors (time in open and closed arms, respectively) (Gomes and Nunes-de-Souza, 2009). By contrast, acute intra-dPAG infusion of 8-OH-dPAT decreased measures of “fear/anxiety” in the RET (e.g., increased post-encounter, neutralized predator, “surface duration”), but not in the MDTB (i.e., no change in *circa*-strike “avoidance distance”) (Pobbe et al., 2011). By contrast, in a separate experiment also employing the MDTB (Griebel et al., 1995), chronic intraperitoneal administration of serotonin reuptake inhibitors (SRIs: imipramine and fluoxetine) decreased the *circa*-strike “avoidance distance.” That is, (1) any reduction in “fear/anxiety” due to intra-dPAG 5-HT_1A_ receptor agonism appears to be specific only to the post-encounter, neutralized predator condition (i.e., only after a potentially threatening external object has been identified), and (2) chronic SRI treatment reduces the “avoidance distance” (i.e., SRIs allow for greater tolerance of closeness in relation to a potential “fight-or-flight” post-encounter, object-relational situation) by a mechanism that is different from the acute 5-HT_1A_ receptor-mediated reduction on post-encounter “fear/anxiety.” Conceivably, SRIs either (1) reduce the rate of production of *circa*-strike “fear/anxiety,” (2) increase the threshold level for the *circa*-strike, “fear/anxiety”-induced emergency alarm, or (3) reduce the gain factor that connects *circa*-strike “fear/anxiety” to the emergency alarm threshold.

Intra-dPAG infusions of CRF (150 pmol/0.2 mL) produced increases in pre-encounter “fear/anxiety” in the EPM (decreased open arm exploration), while separately, infusions of NBI 27914, a CRF-1-receptor antagonist, increased open arm exploration (TOA) (Miguel and Nunes-de-Souza, 2011). In another experiment, The CRF-1 receptor agonist cortagine (100 ng/0.2 mL) increased post-encounter, neutralized predator-induced “fear/anxiety” measures in the RET (decreased “surface duration”) (Litvin et al., 2007). That is, within the dPAG of mice, (1) during pre-encounter exploration, there is tonic CRF-1 receptor agonism by CRF that contributes to generalized “fear/anxiety,” and (2) during post-encounter exploration, the neutralized predator provokes an increase in CRF-induced agonism at CRF-1 receptors, which generates increased “fear/anxiety” and avoidance.

In summary, in the experimental paradigm of Reis et al., prior to “escape” in the rat assay, the rising activity of the “explore” ensemble—which reflects rising post-encounter intrigue with the restrained predatory rat—reaches some critical threshold such that it trips the dPAG countdown timer for the emergency alarm system (Reis et al., 2021a), leading to the increasing activity of the “escape” ensemble, which ultimately overtakes the activity of the “explore” ensemble, triggering the emergency instinctual repression (and motor escape). Conceivably, there is some interaction between the opposing effects of dPAG 5-HT_1A_ receptors and CRF-1 receptors on the surface of the post-encounter “explore”-“fear/anxiety” interface that selectively governs this risky post-encounter ‘interpersonal’ interaction, triggering the emergency psychological repression of the “explore” instinct in these timid and primitive rodents. Perhaps an SRI with additional dual 5-HT_1A_ receptor agonist/CRF-1 receptor antagonist properties would be a useful pharmacological drug to develop for various post-encounter “fear/anxiety”-related conditions of the dPAG. But these considerations also raise the central and specific question as to what, from a psychoanalytic perspective, it is about the “explore” instinct that triggers ego repression in the dPAG of timid mice?

## What makes the “explore” instinct so dangerous such that it must be immediately repressed?

In Freud’s 1926 Conjecture, he stated, “For an instinctual demand is, after all, not dangerous itself; it only becomes so inasmuch as it entails a real external danger…” (Freud, 1926) (p. 126). “We have come to the conclusion that an instinctual demand often only becomes an (internal) danger because its satisfaction would bring on an external danger—that is, because the internal danger represents an external one” (p. 167).

What then would be the phylogenetically adaptive Freudian psychological mechanism that seems to be evolving in mice whereby danger becomes associated with the rising “explore” (intrigue) with the restrained predatory rat? Perhaps in these timid mice, the rising intrigue to explore the presumably neutralized restrained predatory rat ultimately threatens to become, for example, a dangerous rogue “explore” instinct that might impel the mice to act on their desire to enact some ill-advised dominance behavior such as the urge to mark (urinate on) their territory that is still currently being inhabited by the predatory rat (Arakawa et al., 2007). Thus, for evolutionary survival purposes, this potentially rogue “explore” instinct must be censored and immediately and completely repressed. In this situation, it would presumably not be the initially untainted and adventuresome “explore” instinct, *per se*, that is dangerous and provokes emergency repression, but rather it is only upon the acquisition of the associated ill-advised fantasy that attaches itself to this rising “explore instinct” that provokes this necessary and phylogenetically adaptive, post-encounter repression.

## Metapsychological implications: Freudian ego mechanisms in the brainstem

The present considerations add to the accumulating neurobiological evidence (Watt, 1990; Northoff and Boeker, 2006; Watt and Panksepp, 2009; Carhart-Harris and Friston, 2010; Northoff and Scalabrini, 2021) regarding the metapsychological validity of Freud’s Ego-Id model of the mind (Freud, 1923). During his lifetime, Freud could not have known that the neural substrates of the kernel of the ego, including its survival defenses, were housed in the phylogenetically ancient neuronal ensembles in the brainstem dPAG. Fittingly and perhaps prophetically, Freud’s first two publications described the evolutionary implications of the ontogenetic migratory patterns of central nervous system neurons of the phylogenetically ancient larval lamprey (summarized in Freud, 1917, p. 340), an organism whose PAG defensive behavioral functions have been studied for their abiding evolutionary survival importance (Olson et al., 2017; Cisek, 2021; Miyashita et al., 2021). The present computational neuroscience findings of Reis et al. clearly demonstrate that the neuronal ensembles of both instincts as well as ego mechanisms of defense can be housed in the brainstem dPAG. Further, these brainstem instincts can be repressed (suppressed) in the dPAG and rendered subconscious. As such, it appears that the basic Freudian model of the Ego and the Id is in the process of being evolutionarily inscribed into the neuronal hardware and software of the brainstems of vertebrates as far back phylogenetically as mice.

The recent Conscious Id theory of Solms (2013) has threatened to demolish the venerated Freudian Ego-Id model of the mental apparatus. Indeed, Solms (2020) has radically revised (overwritten) Freud’s Project for a Scientific Psychology (Freud, 1950/1895), and has proclaimed that his Conscious Id model of the mental apparatus has turned the ‘theoretical incoherence’ of the ‘classical [Freudian] conception on its head’ (Solms, 2013, p. 12; Solms, 2021b, p. 1047).” Of course, in the present context, one of the central and absolutely necessary pillars upholding Solms’s Conscious Id theory is the claim that Freudian Ego mechanisms cannot possibly be supported by the neural networks of the upper brainstem, and hence that Freudian Ego mechanisms of defense cannot possibly repress and render unconscious any PAG-generated affectively-valenced instincts. “Anybody that knows anything about the upper brainstem will surely agree that it cannot possibly support the functions that Freud assigned to the ego” (Solms, 2022, p. 1177). The results of the present investigation clearly topple this absolutely essential structural pillar of the Conscious Id theory and hence render the whole theory collapsed.

It should be also noted that in Solms’s upgrade of his original Conscious Id model (Solms, 2021a; Solms, 2021b), Solms has reconceptualized and repurposed the 7 emotional operating systems of Panksepp into 7 drives that, for Solms, are now homeostatically regulated according to various PAG set points. However, in his lifetime, Panksepp, the Father of Affective Neuroscience, did not at all regard his 7 emotional operating systems as either homeostatically regulated or even for that matter as drives (Wright and Panksepp, 2012) (p 64). Further, Panksepp famously designated the upper brainstem PAG and its immediate surrounds including the superior colliculi as the neuroanatomic site of the “SELF”—Simple Ego-type Life Form—based on its evolutionarily fundamental role in executing Ego-like survival behaviors (Panksepp and Biven, 2012, p. 415–416; Panksepp, 1998, p. 309). Indeed, Panksepp has stated, “I have chosen to designate such an entity as the SELF (a Simple Ego-type Life Form), and this process may correspond to the most primitive aspect of Freud’s Ego structure” (Panksepp, 1999). For the very same reasons, Merker—whose descriptions of the apparent consciousness of hydranencephlic (“brainstem”) children Solms’s Conscious Id theory relies on so heavily—has also designated this brainstem PAG region as the “Ego-Center” (Merker, 2007).

Hartmann Cardelle, reasoning from pure psychoanalytic theory, mathematical set theory, and cybernetic Mealy Theory (Hartmann Cardelle, 2019), has critiqued Solms’s theoretical assumptions in the Conscious Id formulation and concluded that they based on his oversimplified 1:1 mapping of brain structures onto metapsychological structures; “Hence, the only consistent conclusion that can be drawn from the facts enumerated by Solms and Panksepp (2012) is that the ego emanates from the brainstem, a conclusion that Panksepp has already suggested as early as 1999… In other words, the converging lines of evidence, in conjunction with Freud’s metapsychological definitions, strongly suggest that both the id and the ego originate in the brainstem” (Hartmann Cardelle, 2019). Similarly, Boag (2010), extending his contributions to repression theory, the unconscious, and neuropsychoanalysis, has also noted Freud’s flight-repression hypothesis and has concluded (Boag, 2020), “As such, it is still not precisely clear to me that we have a coherent mechanism of repression in this [Solms’s] revised Project (Solms, 2020). Nevertheless, perhaps the obvious answer here might be in terms of postulating a neural mechanism [of repression]… at the level of the midbrain decision triangle (Merker, 2007) and reticular activating system.”

## Metapsychological implications: Freudian unconscious affects

As noted above, one of the central pillars on which the Conscious Id theory rests is the assertion that there can be no such psychological entity as an unconscious affect. Indeed, Solms has repeatedly and emphatically proclaimed that Freud *insisted* that there are no such thing as unconscious affects (Solms, 2021b) (p. 1045), and therefore that Freud’s formulation that the Id operates according to the pleasure principle is “theoretically incoherent” (p. 1047). Following a challenge to Solms’s assertions (Schwartz, 2022), Solms simply asserted with certainty, “I can reply (as the editor and translator of Freud’s complete works) that, if one studies his writings on this issue in their totality, one is left in no doubt that he [Freud] rejected the notion of unconscious affect, utterly, from first to last” (Solms, 2022) (p. 1174).” However, it is worth noting several notable quotes that span most of Freud’s career (italics added).

“A striking feature in neurotic characters—the fact that a cause capable of releasing an affect is apt to produce in them a result which is qualitatively justified but quantitatively excessive—is to be explained along these same lines, in so far as it admits any psychological explanation at all. The excess arises from sources of *affect which had previously remained unconscious and suppressed*” (Freud, 1900) (p. 479).

“In other words: the distinction between *Cs.* and *Pcs.* has no meaning where feelings are concerned; the *Pcs.* here drops out—and *feelings are either conscious or unconscious*” (Freud, 1923) (p. 23).

“It is familiar ground that the work of analysis aims at inducing the patient to give up the repressions (using the word in the widest sense) belonging to his early development and to replace them by reactions of a sort that would correspond to a psychically mature condition. With this purpose in view *he must be brought to recollect certain experiences and the affective impulses called up by them which he has at the moment forgotten*… Again, he produces ideas, if he gives himself up to ‘free association’, in which we can discover allusions to the repressed experiences and derivatives of the *suppressed affective impulses* as well as of the reactions against them. And, finally, there are hints of repetitions of the *affects belonging to the repressed material* to be found in actions performed by the patient, some fairly important, some trivial, both inside and outside the analytic situation” (Freud, 1937) (pp. 257–8).

Thus, it is quite clear that—notwithstanding Solms’s confident proclamations—Freud certainly did not *insist*, from first to last, that affects were necessarily and exclusively conscious entities. Thus again, this one consideration alone also is sufficient to topple Solms’s Conscious Id theory.

As such, the results of all of these present computational neuroscience, neuropsychoanalytic, and metapsychological considerations indicate that Solms’s Conscious Id theory commits theoretical violence and leads to the inescapable conclusion that affectively-valenced Freudian instincts can be subject to repression and rendered subconscious by Freudian ego mechanisms in the dPAG—thereby conclusively invalidating the briefly influential but ultimately misguided challenge by Solms’s Conscious Id model to Freud’s Ego-Id model. The phylogenetic durability of the Freudian Ego-Id model is therefore conclusively demonstrated.

## Clinical implications: psychotherapy and psychoanalysis

Perhaps the most consistently cited upgrade to psychotherapy and psychoanalytic technique that has been said to result from adoption of the Conscious Id metapsychological model is that truly unconscious, non-declarative (“illegitimately automatized”) memories can never be retrieved, and hence “deep” memory recovery should not be pursued in psychotherapy and psychoanalysis. That is, instead of trying to recover repressed memories, the therapist/analyst should work to develop transference repetitions, such that better and smarter cognitive solutions can then be offered to the patient for his/her permanently inaccessible and insoluble childhood problems. This essential modification of psychotherapeutic technique that is derived from the Conscious Id theory has been echoed and developed by others (Flores Mosri, 2018; Balchin et al., 2020). Thus, according to Solms (2018):

“Where I differ from Freud in this regard is that *I do not believe that the repressed ever returns*; it is only the *affect*, which it fails to regulate, that returns…Normally, in order for predictions to be updated, in light of experience, they need to be reconsolidated; that is, they need to enter consciousness again, in order for the long-term traces to become *labile* once more. This is impossible to achieve for repressed predications, because the essential mechanism of repression entails immunity from reconsolidation, despite prediction errors…The pathogenic predictions cannot be remembered directly for the very reason that they are automatized (i.e., non-declarative)… Reconsolidation is thus achieved through activation of non-declarative traces via their *derivatives in the present* (this is called “transference” interpretation). Therefore, the analyst identifies them indirectly by bringing to awareness the *repetitive patterns of behavior* derived from them… The unconscious is just that: it is unconscious forever more. Although we can infer it, we can never experience it, such inferences (called “reconstructions in psychoanalysis”) help us to better understand the here and now transference. On the basis of this understanding, all we can hope to achieve is new and better predictions which must be consolidated alongside the old ones.”

Now contrast Solms’s modern neuropsychoanalytic therapeutic innovation based on his Conscious Id model with what Freud said in “Constructions in Analysis” (Freud, 1937):

“We know that his present symptoms and inhibitions are the consequences of repressions of this kind: thus, that they are a substitute for these things that he has forgotten. What sort of material docs he put at our disposal which we can make use of to put him on the way to recovering the lost memories? All kinds of things… Our experience has shown that the relation of transference, which becomes established toward the analyst, is particularly calculated to favor the return of these emotional connections… What we are in search of is a picture of the patient’s forgotten years that shall be alike trustworthy and in all essential respects complete… What then *is* his [the analyst’s] task? His task is to make out what has been forgotten from the traces which it has left behind or, more correctly, to *construct* it… How this occurs in the process of the analysis—the way in which a conjecture of ours is transformed into the patient’s conviction—this is hardly worth describing. All of it is familiar to every analyst from his daily experience and is intelligible without difficulty. Only one point requires investigation and explanation. The path that starts from the analyst’s construction ought to end in the patient’s recollection; but it does not always lead so far. Quite often we do not succeed in bringing the patient to recollect what has been repressed. Instead of that, if the analysis is carried out correctly, we produce in him an assured conviction of the truth of the construction which achieves the same therapeutic result as a recaptured memory. The problem of what the circumstances are in which this occurs and of how it is possible that what appears to be an incomplete substitute should nevertheless produce a complete result—all of this is matter for a later enquiry” (Freud, 1937).

Thus, it seems that the main clinical recommendation that Solms has devised is that we should avoid digging too deeply for unconscious memories. However, as Solms himself has stated, although unconscious memories never return, “…it is only the *affect*, which it [repression] fails to regulate, that returns.” Solm’s contribution to neuropsychoanalysis has certainly been an important one. Effective cognitive work and consolidation should be an important element of any psychoanalytic treatment (Kernberg, 1988). As Freud noted, it is quite legitimate to ask, how much repressed memory can and should the clinician try to recover in the patient for the purposes of making dynamic sense out of, and resolving, their patients’ current life conflicts and deficits? Regardless, such considerations do not in any way justify or require some newfangled metapsychological model to replace Freud’s tripartite model.

## Neuropsychoanalysis has become much more than just the Conscious Id

The present demonstration of Freudian Ego defense mechanisms in the upper brainstem reinforces the conviction that neuropsychoanalysis will continue to (1) enhance the clinical and scientific legitimacy of psychoanalysis around the world, and (2) make further important contributions to the neurobiology of other metapsychological substrates, such as instinct, defense, memory systems, consciousness, interoception, ego flexibility, and many others. These important lines of neuropsychoanalytic research will undoubtedly continue without the Conscious Id model.

## Summary

In “The Ego and the Id,” Freud stated, “We thus obtain our concept of the unconscious from the theory of repression. The repressed is the prototype of the unconscious for us… We have formed the idea that in each individual, there is a coherent organization of mental processes; and we call this his ego… From this ego proceed the repressions, too, by means of which it is sought to exclude certain trends in the mind not merely from consciousness, but also from other forms of effectiveness and activity… Moreover, the ego seeks to bring the influence of the external world to bear upon the id and its tendencies; and endeavors to substitute the reality principle for the pleasure principle which reigns unrestrictedly in the id” (Freud, 1923).

**Figure 2:**
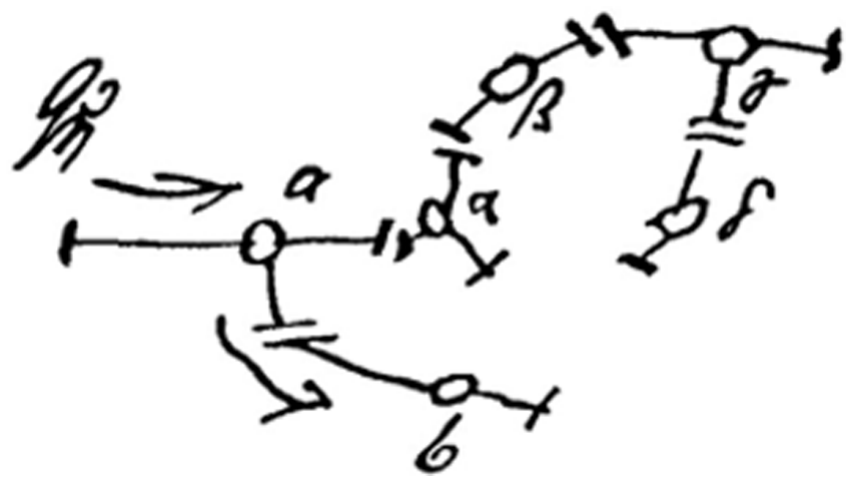
The kernel of the Freudian Ego circa 1895. Freud described his hypothetical prototypical neuronal ensemble that executes ego repression as follows, “Let us picture the ego as a network of cathected neurones well facilitated in relation to one another... If we suppose that a Qή enters a neurone a from outside (ϕ), then, if it were uninfluenced, it would pass to neurone *b*; but it is so much influenced by the side-cathexis *a*-α that it gives off only a quotient to *b* and may even perhaps not reach *b* at all. Therefore, if an ego exists, it must *inhibit* psychical primary processes.” Reproduced from Freud, (1895). *A Project for a Scientific Psychology. Standard Edition*, 1:324, according to the Library of Congress public rights access statement to the Freud archives.

“For an instinctual demand is, after all, not dangerous in itself; it only becomes so inasmuch as it entails a real external danger…” (Freud, 1926). “If the ego succeeds in protecting itself from a dangerous instinctual impulse, through for instance, the process of repression, it has certainly inhibited and damaged the particular part of the id concerned; but it has at the same time given it some independence and has renounced some of its own sovereignty. This is inevitable from the nature of repression, which is, fundamentally, an attempt at flight. The repressed is now, as it were, an outlaw; it is excluded from the great organization of the ego and is subject only to the laws which govern the realm of the unconscious” (Freud, 1926; Figure 2).

Freud’s far-reaching 1926 conjecture regarding the phylogenetic origin of ego defense appears confirmed, and the location of Freud’s prototypical ego defense is the dPAG. These evolutionary considerations may open the door for future psychological, psychoanalytic, neuropharmacological, and neurobiological investigations of some of the most debilitating and treatment-refractory behavioral and emotional disorders of the dPAG emergency alarm system. Further studies that could prove beneficial include, (1) the interactions between dPAG 5-HT, 5-HT_1A_ receptors, and CRF-1 receptors that regulate “fear/anxiety” and “avoidance distance” for interpersonal (post-encounter) relationships, and (2) the psychoanalytic characterization and treatment of patients with pharmacologically treatment-refractory panic and separation disorders as well as the many other conditions that are probably associated with aberrant operation of the dPAG emergency alarm system. These studies become particularly relevant as people are becoming increasingly debilitated in a world where dangerous threats, violence, disavowal of personal responsibility and guilt, and recalcitrant belief systems have become a routine part of our interpersonal, social, and political fabric (Schwartz, 2016).
